# Detection of Freezing of Gait in Parkinson Disease: Preliminary Results

**DOI:** 10.3390/s140406819

**Published:** 2014-04-15

**Authors:** Christine Azevedo Coste, Benoît Sijobert, Roger Pissard-Gibollet, Maud Pasquier, Bernard Espiau, Christian Geny

**Affiliations:** 1 INRIA-LIRMM, Montpellier 34095, France; E-Mail: benoit.sijobert@inria.fr; 2 INRIA, Montbonnot 38330, France; E-Mails: roger.pissard@inria.fr (R.P.-G.); pasquier@gmail.com (M.P.); espiau@inria.fr (B.E.); 3 CHU, Montpellier 34295, France; E-Mail: c-geny@chu-montpellier.fr

**Keywords:** freezing of gait (FOG), festination, inertial measurement units, gait parameters, Parkinson's disease

## Abstract

Freezing of gait (FOG) is a common symptom in Parkinsonism, which affects the gait pattern and is associated to a fall risk. Automatized FOG episode detection would allow systematic assessment of patient state and objective evaluation of the clinical effects of treatments. Techniques have been proposed in the literature to identify FOG episodes based on the frequency properties of inertial sensor signals. Our objective here is to adapt and extend these FOG detectors in order to include other associated gait pattern changes, like festination. The proposed approach is based on a single wireless inertial sensor placed on the patient's lower limbs. The preliminary experimental results show that existing frequency-based freezing detectors are not sufficient to detect all FOG and festination episodes and that the observation of some gait parameters such as stride length and cadence are valuable inputs to anticipate the occurrence of upcoming FOG events.

## 1. Introduction

Parkinson's disease (PD) is the second most common neurodegenerative disorder, and according to the Parkinson's Disease Foundation, 10 million people worldwide are affected [1,2]. This chronic disease can lead to gait disturbances and falls which cause an important reduction of the quality of life. Gait in PD patients is characterized by a reduction in step length and velocity, decreased angular displacement and velocity of lower and upper limbs, high variability of step timing, poor bilateral coordination and asymmetric leg function [3]. A prospective 20-year follow-up of PD patients reported a high prevalence of falls (87%) and fractures (35%) [4]. The pathophysiology of falls in PD is complex and multifactorial. Falls occurring in patients with advanced Parkinson's disease can be related to a particular paroxysmal symptom called freezing of gait (FOG). FOG is defined by Nieuwboer and Giladi [5] as “an episodic inability (lasting seconds) to generate effective stepping in the absence of any known cause other than Parkinsonism or high-level gait disorders”. It can occur during initiation of the first step, turning [6], dual tasks, walking through narrow spaces, reaching destinations or passing through doorways [7,8]. FOG episodes are more often brief (1–2 s), but can also last 10 s. They are reported by the patient as a subjective feeling of “the feet being glued to the ground”. Festination while walking, another symptom of Parkinsonism, is defined clinically as a tendency to move forward with increasingly rapid, but ever smaller steps, associated with the center of gravity falling forward over the stepping feet [9]. The relation between festination and FOG is an important issue, which is not always well described in the literature. Focused attention and external stimuli (cues) can help to overcome a FOG episode [10]. It is well known from clinicians and patients that auditory rhythmic stimulation or visual marks on the ground improve dramatically gait in patients with FOG. This sensibility to cueing has led some teams to develop mobility aid devices: cane with a laser light visual cue or visual auditory walker [11,12].

Clinical assessment of FOG episodes remains difficult and the impact on the daily life is generally assessed with validated questionnaires [8,13]. In the majority of the studies, a careful quantification of gait abnormalities is performed with various movement assessment techniques. They have been especially used to demonstrate the impact of cueing on gait characteristics [14]. However, large rehabilitation programs have failed to confirm a long lasting effect. Functional Electrical Stimulation (FES) has also been tested and preliminary results show FOG reduction observed during FES-assisted gait of PD patients [15,16].

Clinical evaluation of video recordings of patients by one to three observers is the gold standard to identify FOG events [17]. The evaluation of clinical effects of the treatments would benefit from objective, standardized FOG measures [18]. Moore *et al.* have proposed a technique to identify FOG episodes based on the frequency properties of leg vertical accelerations. The approach is based on the hypothesis that FOG occurrences are associated to trembling motion, which affect limb acceleration signal. They have introduced the so-called freeze index (FI): the ratio between the signal (limb acceleration) power in the “freeze” band and the signal power in the “locomotor” band. The FI method was validated using one to seven accelerometers mounted on patients with satisfactory detection results [19]. In the present paper we propose a complementary index in order to take into account both trembling and festination situations. Our objective is to anticipate the occurrence of FOG episodes in order to propose a robust solution for real-time control of assisted devices. We believe, festination is one of the FOG expressions and precedes most of gait interruptions and is an interesting marker of gait modification. Furthermore we intend to propose a solution based on a minimal number of embedded sensors and detection algorithms for future real-time applications.

In Section 2 we recall the definition of FI and introduce our FOG criterion (FOGC). In Section 3 we present the inertial sensor on which our solution is based and we describe the experimental setup. In Section 4 the results are described and discussed.

## 2. Freezing of Gait Detection

Moore *et al.* introduced the freeze index (FI) as the power of the considered body segment acceleration signal in the “freeze” band (3–8 Hz) divided by the power of the signal in the “locomotor” band (0.5–3 Hz) [18–20]. For each instant t, FI(t) is defined as the square of the area under the power spectra of a 6 s window of data (centered at time t) in the “freeze” band, divided by the square of the area under the power spectra in the “locomotor” band. The width of the sliding window is based on FOG duration. It has been determined that the optimal window width has to approximately be twice the duration of the shortest FOG event to be detected. Increasing the window size reduces the sensitivity of the FI, acting as a low-pass filter by not identifying the short-duration freeze events. A FI threshold is chosen such that FI values above this limit are designated as FOG. In their article, Moore *et al.* have chosen the threshold as the mean plus one S.D. of the peak FI from nine epochs of volitional standing.

Here, we introduce a new approach for the observation of gait changes and the detection of FOG events, the so-called FOG criterion (FOGC). FOGC is based on the continuous evaluation of two gait parameters: cadence and stride length. Cadence during festination in PD patients reported values less than 3 Hz (2.8 Hz [S.D.0.2]) [5]. Our hypothesis is that, before a FOG event occurs, the cadence should increase whereas the stride length decreases (festination).

For each detected stride *n*, its frequency (cadence) is denoted *C_n_*, its length *L_n_*, and *FOGC_n_* the related following criterion:

$$FOGC_{\text{n}}=\frac{C_{n▪}\;L_{\text{min}}}{C_{\text{max}▪}(L_{n}+L_{\text{min}})}$$

where *C_max_* and *L_min_* are the expected maximal value for the cadence and minimal value for the length of strides, respectively. A minimum step length of *L_min_* = 5 cm can be observed in some patients. Thus, in order to bound the criterion to 1 when stride length tends to 0, the maximum cadence has been fixed to *C_max_* = 5 strides/s for compensating the values. The gait cycle segmentation used does not detect strides below 1/*C*_max_ duration. A high value of the FOGC is associated to a freezing of gait event. A criterion increase should indicate an imminent FOG episode.

Like the FI, the FOGC value needs to be compared to a threshold adjusted individually for each patient. Besides, the criterion being linked to gait parameters, it only allows festination and FOG event detection during gait cycle. The gait segmentation and the stride length calculation have been performed using [21] inertia sensor-based walking speed estimation methods. This method is based on the segmentation of gait data into strides using gyroscopic data. Within each stride the acceleration data is integrated in order to obtain the forward leg displacement. The initial velocity of the leg at the stride onset is obtained using gyroscopic signal. At the end of the stride, a correction is performed between the velocity estimated using accelerometric data and the values measured by gyroscopic sensors. Additionally, a homogeneous transformation is performed to project sensor's measures into the sagittal plane.

## 3. Experimental Section

**Figure 1.**
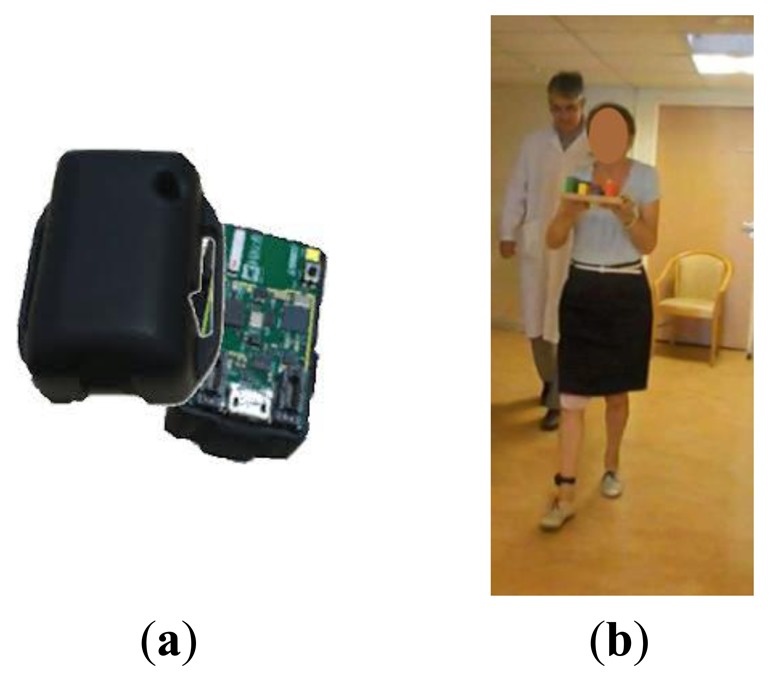
(**a**) FOX node: wireless inertial measurement unit. (**b**) Patient equipped with the sensor.

**Figure 2.**
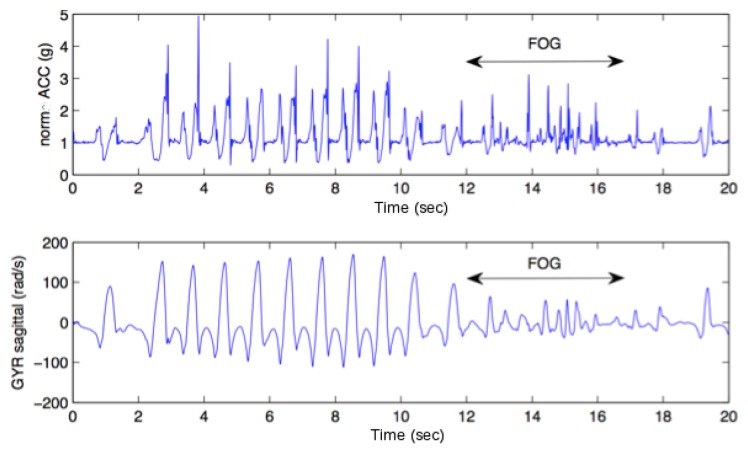
Example of FOG episode observed through an IMU sensor.

The motion capture system is based on a HikoB Fox^©^ (Villeurbanne, France) (Figure 1). This node is an inertial measurement unit (IMU): ultra compact, ultra low power and wireless. It has three main functionalities: acquisition of inertial data, data processing based on a 32 bits micro-controller (STM32 by STMicroelectronics^©^, *Geneva, Switzerland*) and wireless 2.4 GHz radio-frequency communication (802.15.4 PHY standard). The motion capture acquisition consists of a 3D accelerometer, a 3D magnetometer and a 3D gyrometer whose data is stored on a micro SD card at a frequency of 100 Hz. The data synchronization acquisition with several nodes is possible using a radio beacon. IMU sensors are well adapted to detect gait pattern changes like FOG episodes (Figure 2).

A video camera was synchronized with the inertial sensor. The patient gait was analyzed offline based on the video recordings. A clinician spotted FOG events and classified them as follows: (1) slight modification of the gait with no falling risk (green); (2) main gait modification with falling risk (orange); (3) FOG gait is blocked with or without festination (red). Local ethical committee approved the study.

Four patients, (males, 73 ± 3 years old) participated to this preliminary study. The inertial sensor was placed at the shank level in the sagittal plane (Figure 1). The patients were asked to walk along a 10 m corridor and several dual tasks were proposed in order to maximize the number of FOG occurrences.

## 4. Results and Discussion

**Figure 3.**
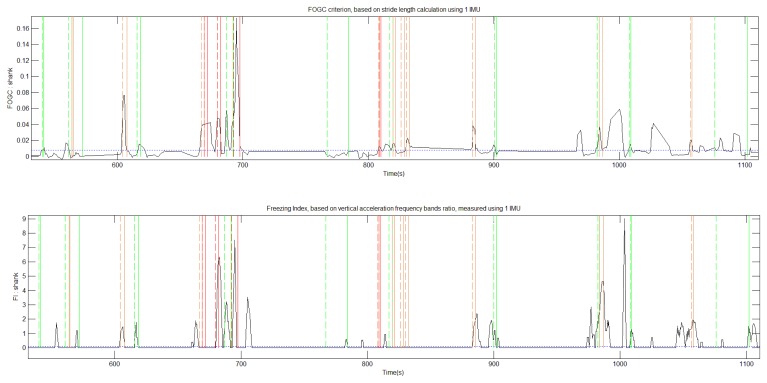
FOGC (**Top**) and FI (**Bottom**) were calculated from inertial measures provided by one IMU placed at ankle. Based on video recordings, the FOG events were classified from green to red (vertical lines), regarding their intensity. A FOG detection threshold is set to 0.008 for the FOGC and 0.1 for the FI (horizontal blue lines), with a 4 s NFFT window.

**Figure 4.**
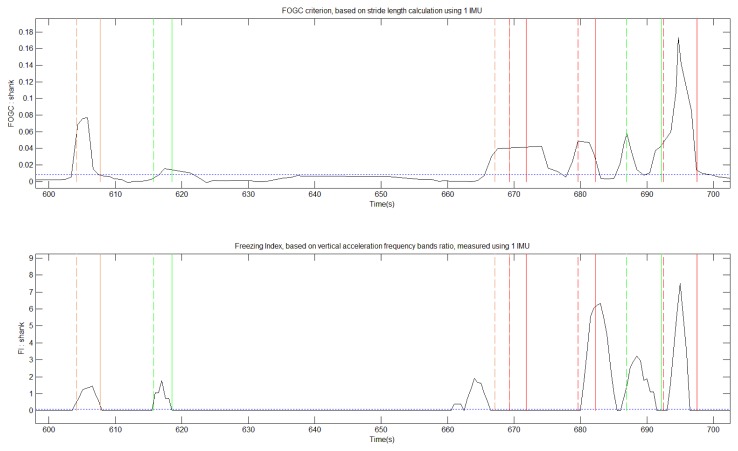
Zoomed view of Figure 3. Around 670 s, an important FOG event is detected by FOGC (**Top**) but not FI (**Bottom**).

**Figure 5.**
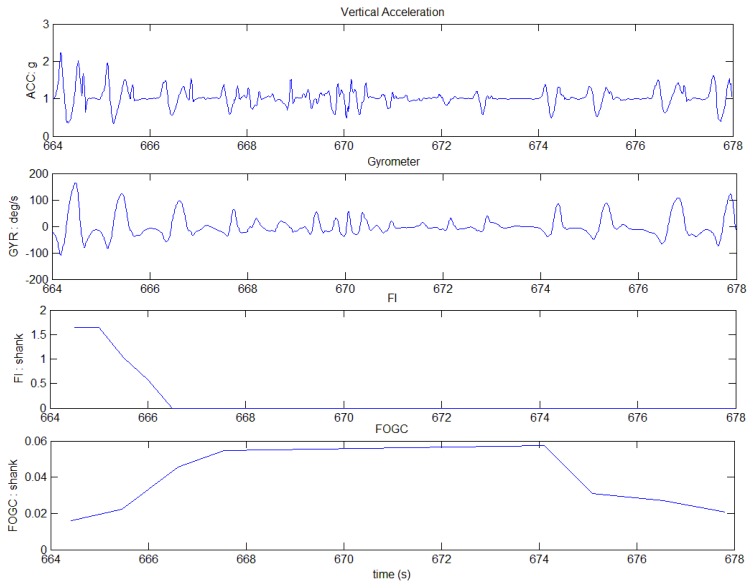
IMU rough data corresponding Figure 4 non-detected FOG event.

IMU signals and video were recorded during 1,730 s. Video from the sessions were analyzed by the neurologist who identified and labeled 44 FOG episodes (Table 1). IMU sensor data was processed in order to compute FI and FOGC indexes (Figure 3). The FI method detected 26 of these episodes and the FOGC method detected 35 of these episodes. Concerning the 26 main FOG events (labeled red and orange), FI “missed” nine FOG events and FOGC missed only four of them (Figures 4 and 5).

These observations emphasize the FOG heterogeneous characteristics and show that FI method is not able to detect all the episodes. Additionally, from the videos, we were able to clearly identify three festination episodes preceding FOG events. None of them was detected by the FI method whereas FOGC detected all of them. Due to its definition FI is not sensitive to festination as the method uses power frequency band ratios observed on vertical acceleration changes.

## 5. Conclusions/Outlook

Freezing and festination of gait in Parkinson's Disease are associated with a high fall risk. Objectively detecting FOG and festination episodes during the daily activities of a patient would allow to better assess a patient's state and the clinical effects of treatments. Furthermore real-time detection of those FOG events could allow triggering assistive/cueing devices at the right time. Indeed, if visual or auditory aids can help patients to reduce freezing occurrences, the efficiency decreases within time. Triggering the systems only when needed may be a solution to reduce the habituation effect.

FI index proposed by Moore *et al.* detects changes in the power spectra of inertial signals and makes it possible to detect FOG events. IMU data allow going further in the analysis of gait by estimating various parameters. The interpretation of data is more intuitive as the variables considered can be linked to walking observations. We demonstrated here that stride length and cadence could for instance be interesting to observe in order to detect gait changes including FOG and festination. In the future we will extend the approach by observing other gait parameters and perform experiments on a larger population in order to validate our method. We believe the FI index could be associated to methods based on gait parameters in order to detect walk patterns modification and possibly anticipate the occurrence of a FOG event.

## Figures and Tables

**Table 1.** FOG episodes detected through video analysis, via FI and FOGC algorithms.

**FOG Intensity**	**Video**	**FI**	**FOGC**
Green	18	9	13
Orange	12	6	10
Red	14	11	12
False positives	0	17	13
